# How Biochar Derived from Pond Cypress (*Taxodium Ascendens*) Evolved with Pyrolysis Temperature and Time and Their End Efficacy Evaluation

**DOI:** 10.3390/ijerph191811205

**Published:** 2022-09-06

**Authors:** Shuai Zhang, Haibo Hu, Xiangdong Jia, Xia Wang, Jianyu Chen, Can Cheng, Xichuan Jia, Zhaoming Wu, Li Zhu

**Affiliations:** 1 College of Forestry, Nanjing Forestry University, Nanjing 210037, China; 2 Co-Innovation Center of Sustainable Forestry in Southern China, Nanjing 210037, China; 3 College of Science, Nanjing Forestry University, Nanjing 210037, China; 4 Wuxi Branch, Bureau of Investigation on Hydrologic Water Resources, Wuxi 214100, China

**Keywords:** pond cypress (*Taxodium ascendens*), branch-based or leaf-based biochar, pyrolysis temperature and time, the principal component and cluster analysis

## Abstract

Biomass type, pyrolysis temperature, and duration can affect biochar properties simultaneously. To further clarify the mechanism of this interaction, the branch and leaf parts of Pond cypress (*Taxodium ascendens*) were separately pyrolyzed at four peak temperatures (350 °C, 450 °C, 650 °C, and 750 °C) for three different durations (0.5 h, 1 h, and 2 h) in this study. The resulting biochar properties were measured, which included the yield, specific surface area (SSA), pH, EC (electricity conductivity), the bulk and surface elemental composition, and the contents of moisture, ash, fixed carbon, and volatile matter. The results showed that the pyrolysis temperature was more determinant for the modification of all biochar, but the residence time had a significant effect on the yield, pH, and SSA of branch-based biochar (B-biochar) at specific temperatures. However, such a phenomenon only happened on the pH of leaf-based biochar (L-biochar). Results: (1) With the temperature at 350 and 650 °C, the residence time had a significant effect on the yield of B-biochar. (2) The pH of B-biochar and L-biochar varied considerably between durations when the heating temperature hit 650 and 750 °C. (3) The SSA of B-biochar possessed an obvious fluctuation with the time during the pyrolysis from 650 to 750 °C. According to the properties measured above, the principal component and the cluster analysis classified the 24 types of biochar made in this experiment into four groups and revealed that an obvious disparity existed between B-biochar and L-biochar that were pyrolyzed at temperatures ranging from 450 to 750 °C, which suggested that biomass type was the primary factor for biochar-making. All this information can provide valuable references for the optimization of biochar-making in the real world.

## 1. Introduction

Biochar is a by-product of biomass (usually forestry, agriculture, and chemical residuals or wastes) via thermochemical modification [1,2,3], gasification [4,5], hydrolysis [6], and pyrolysis [7]. Despite these diverse techniques for biochar-making, the technology of direct pyrolysis is more convenient, cost-effective, and ecologically friendly [8,9,10]. Due to the dehydration, decarboxylation, and demethylation during pyrolysis, biochar usually has a higher pH and electrical conductivity (EC), which can adjust soil pH, enhance soil cation exchange capacity (CEC), and promote nutrient supplementation in soil for plant growth [11,12,13]. Also, thermal treatment can convert the organic carbon of raw materials into stable aromatic carbon structures. This means that carbon in biochar can be sequestered to decrease greenhouse gas emissions and then mitigate global warming [13,14,15,16]. Biochar has also been widely used in water treatment and soil remediation in recent years. That is because its fine porous structure and great specific surface area (SSA) enhance the adsorption of pollutants, e.g., heavy metal ions or organic contaminants, in soil or water [3,17,18,19,20].

Therefore, the potentials of biochar in future applications are closely related to its properties, like the pH, EC, and SSA that were mentioned above, which are mainly determined by biomass type and pyrolysis conditions [15,21,22,23,24,25]. The properties of biochar vary significantly depending on the raw material species. For example, after pyrolysis of four feedstocks (including oak, pine, sugarcane, and peanut shell) at 350–900 °C, the pH and EC of the resulting biochar varied considerably with feedstock type, but the yield between feedstocks varied less significantly, which is more determined by the temperature [22]. When biochar was derived from three feedstocks (Douglas fir wood, Douglas fir bark, and poplar wood) by pyrolysis at the temperature range from 350 to 600 °C, their pH and EC still varied significantly among feedstocks, and the biochar yield fluctuated by about 10% between raw materials [23]. However, current studies on the source material of biochar-making have been confined to the disparity among species, with less attention to the possible differences between biochar derived from the same species, such as different parts of the same tree species.

Among research on pyrolysis conditions, the effects of heating temperature have been the focus. Its determinant role in biochar modification has been testified by the considerable variations of biochar characteristics on thermos-sequences. Some of the patterns with the pyrolysis temperature are as follows: the pH value, EC, fixed carbon content, aromatic degree, and specific surface area of biochar significantly increase with the pyrolysis temperature, while the oxygen, hydrogen content, as well as the yield decrease substantially. At the same time, the contents of moisture, volatile matter, and ash fluctuate [7,9,15,21,22,23,24,25,26,27]. Nonetheless, the residence time of pyrolysis can be another important factor in biochar modification [15,28,29,30,31]. Zornoza claimed that the longer the residence time during pyrolysis, the less labile organic the matter contained in biochar [12,15]. Moreover, in 2014, Shaaban reported that the specific surface area of biochar fluctuated as holding time lasting from 1 to 3 h. There was an increase from 1.8 to 1.9 m^2^·g^−1^ at 300 °C but a drop from 2.7 to 1.9 m^2^·g^−1^ at 500 °C [29]. By contrast, Cross and Sohi in 2013 suggested that no remarkable effects from holding time were exerted on biochar stability at a high-temperature range [30]. Zhang in 2015 also pointed to an absence of significant effects on biochar properties for heating time [31]. The inconsistency among studies has indicated intricate effects of residence time, which require more investigation due to its indispensable role in the pyrolysis of biochar-making in the real world.

Based on the current knowledge, two assumptions were put forward in advance. First, biochar derived from different parts (branch or leaf part) of the same tree species may have significantly different characteristics. Second, the pyrolysis temperature and source material type may limit biochar characteristics with the residence time. As a result, the current study aimed to (a) identify how biochar from branch and leaf parts of pond cypress (*Taxodium Ascendens*) would evolve with the pyrolysis temperature and time; (b) clarify the relationship between effects from the pyrolysis temperature, residence time and source material characteristics; (c) classify and evaluate all manufactured biochar according to properties they performed; and (d) make a comparison between biochar from the pond cypress and other tree species.

In addition, the biomass in this study would be the pond cypress (*Taxodium ascendens*), a common coniferous tree species in southern China with its propensity for deep, loose, damp, acidic soil. It is also fast-growing, cold-hardy, resistant to water and humidity, and quite resilient to drought. These features make it an important tree species for gardening and reforestation in the north and south water network areas along the Yangtze River. In the border area between Jiangsu and Anhui alone, more than a few national wetland parks boast forests with pond cypress as the main species.

## 2. Materials and Methods

### 2.1. Biochar Collection

The biomass feedstock pond cypress (*Taxodium ascendens*) was from the Chi Shan Lake Park (118°41′ 28.362″ E,32° 22′ 59.175″ N), the national wetland park located at the junction area of Liuhe District, Nanjing City, Jiangsu Province and Laian County, Chuzhou City, Anhui Province. The park boasts an aquatic forest with more than 50,000 artificially planted pond cypress trees.

Different parts of the biomass, including branches and foliage, were washed, cut, and divided into two heaps, one for branch part and the other for foliage part. Then they were dried at 105 °C for 24 h before ground into powder under 0.22 mm using a stainless-steel screen for further pyrolysis. According to a National Renewable Energy Laboratory (Golden, CO, USA) protocol (the NREL method) [32], the composition of lignin, cellulose, and semi-cellulose feedstock was analyzed (Table 1).

### 2.2. Biochar Preparation

In a laboratory quartz tube furnace, the ground raw material powder (branch, leaf separately) contained in square crucibles with lids got pyrolyzed at four different temperatures: 350, 450, 650, and 750 °C; and set three heating times for each temperature: 0.5, 1, and 2 h. High purity nitrogen was continuously purged into the furnace tube to ensure an oxygen-free condition, with the heating rate fixed at 5 °C per minute for each run. After being cooled to room temperature (approximately 20–25 °C), the carbonized samples were taken out and immediately weighed for later yield analysis. There were at least three repeated aliquot portions for each temperature and holding time. The finally generated biochar was combined, mixed, and mildly milled to pass 200 mesh sieves before being sealed in an air-tight container for later analysis and experiments. B-biochar (biochar from branches) and L-biochar (biochar from foliage or leaf part) were used to mark these biochar samples.

### 2.3. Biochar Yield

After carbonization, the output rate of biomass is referred to as the biochar yield [33]. Biochar yields in this study are reported as a percentage of the weight of the dry feedstock and are presented as feedstock recovery [22]. The calculation is shown in following equation:

$$the\;yield\;\left(\%\right)=\frac{W_{b}}{W_{0}}\times 100$$

where $W_{0}$ is the weight of the dry raw material before pyrolysis, while $W_{b}$ is the weight of carbonized biochar [23,31].

### 2.4. The pH, Electrical Conductivity (EC) and Specific Surface Area (SSA) of Biochar

1 g for the subjected feedstock and biochar were selected and thoroughly mixed with deionized water at the ratio of 1:20 (m·v^−1^) [3]. After being allowed to equilibrate for 1 h, the suspensions were measured for both pH and EC using a pH meter Mettler Toledo Fe 20 and an EC meter Mettler Toledo Fe 38. Both the analyses of pH and EC were performed in triplicate. Brunauer–Emmett–Teller (BET) method with carbon dioxide (CO_2_) adsorption at 0 °C were used to measure the specific surface area (SSA) of the biochar. The CO_2_ adsorption was carried out by BET measurement instrument (Autosorb-IQ-MP, Quanta chrome, Boynton Beach, FL, USA).

### 2.5. Proximate Analysis

Proximate analysis was carried out according to a modified American Society for Testing Materials method (ASTM D1762-84). Moisture was determined by calculating the weight loss after heating samples at 105 °C for 24 h. After that, the samples were heated in a high-temperature muffle furnace (Fisher Scientific, Waltham, MA, USA) at 900 °C for 6 min, during which the weight loss was the volatile matter (VM). Then the ash content was determined by the weight of the residues after heating samples at 750 °C for 6 h. At last, fixed carbon (FC) was calculated by the difference in moisture, volatile matter (VM), and ash contents [3,22,23].

### 2.6. Elemental Composition Analyses

Elemental composition analyses included two processes. The first is ultimate analysis referring to the measurement of C, N, H, and O contents in bulks of raw materials and all manufactured biochar. Contents for C, N, and H got determined by using the elemental analyzer, PerkinElmer 2400 II USA, while the O content was determined by combusting the sample at 1150 °C using another elemental analyzer, Elementar Vario EL III Germany. The measure results were used to calculate the molar H/C and O/C ratio, which are indicative of the stability of C structure in biochar. The second is the measurements of S, P, K, Ca, Mg, Na, and Si contents on the biochar surface using the energy-dispersive X-ray spectroscopy (INCA X-ACT Oxford Instruments, Oxford, England). Before measurement, all subjected samples were dried at 80 °C for more than 24 h.

### 2.7. Statistics

Difference between biochar samples were analyzed through two-way ANOVA, with a significance level of 0.05. Pearson’s correlation analysis was carried out for the relationship between the measured parameters. Factor analysis was performed for all biochar properties determined in this study to distinguish important components in various biochar properties. Due to the strong correlation between the ultimate analysis, the proximate analysis, and the biochar surface element analysis, a factor analysis was performed to reduce the dimensionality of indicators in these analyses into several principal components. Based on the resulting principal components plus the yield, and the specific surface area of biochar, cluster analysis was performed to classify biochar into different groups using the hierarchical clustering method. All statistical analyses were conducted using IBM SPSS (IBM, Armonk, NY, USA) and Origin Pro 2021 (Origin Lab, Northampton, MA, USA) statistics, which were available as learning trials through the official platform.

## 3. Results

### 3.1. The Biochar Yield

**Figure 1:**
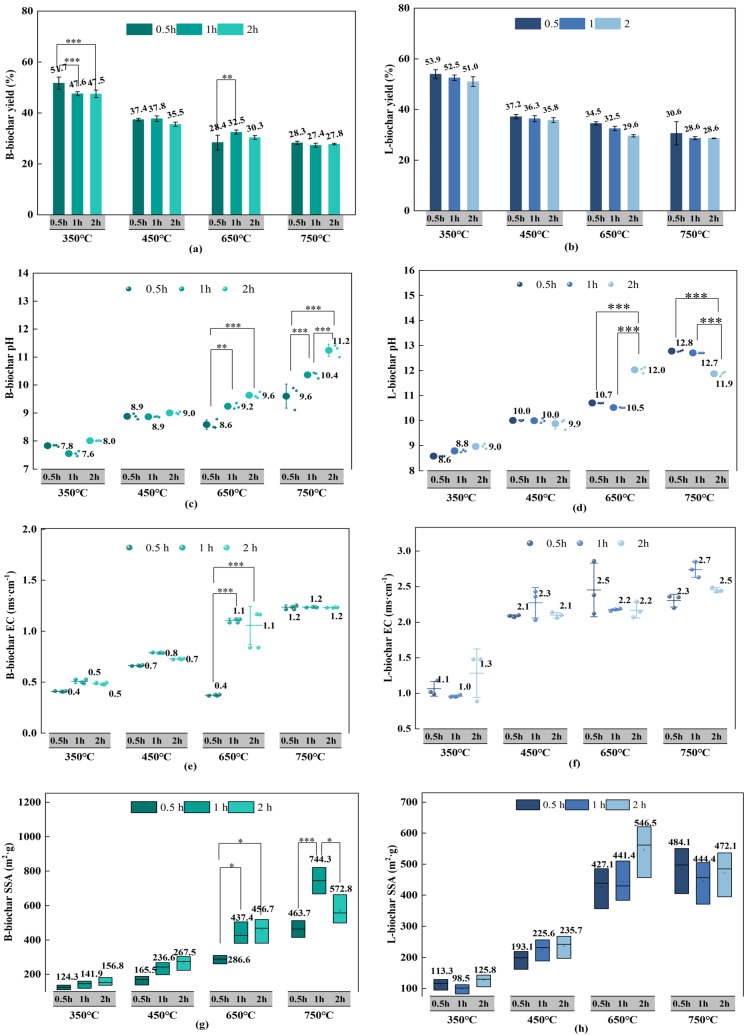
An illustration of how the yield, Ph, EC, and SSA of all manufactured biochar evolved with the time when the temperature was constant: (**a**,**c**,**e**,**g**) respectively indicate the yield, pH, EC and specific surface area (SSA)of B-biochar; while (**b**,**d**,**f**,**h**) indicate these properties of L-biochar; the significance was noted by * = *p* < 0.05, ** = *p* < 0.01 and *** = *p* < 0.001.

B-biochar and L-biochar made from pond cypress (*Taxodium Ascendens*) showed a decreasing yield as the pyrolysis temperature rose (Figure 1a,b). However, the biochar yield with time was variable. Therefore, the evolution with time is the focus to note. Figure 1a shows a drastic decline of about 4% in biochar yield when B-biochar pyrolyzed from 0.5 to 1 h at 350 °C. At 650 °C, biochar pyrolyzed for 0.5 h attained a yield of about 32.5%, 4% higher than the yield for 1 h of pyrolysis. However, no significant fluctuations between durations was found when the temperature was at 450 °C and 750 °C. By contrast, the biochar yield of L-biochar decreased gradually over time at four temperature settings, with its lowest rate of 28.6% at 750 °C for 2 h (Figure 1b). These performances were in line with the results of the two-way ANOVA: pyrolysis temperature and time affected the yield of B-biochar in a combined way, but this interactive effect did not exist in L-biochar yield. In addition, biochar made from pond cypress (*Taxodium ascendens*), irrespective of the feedstock branch or leaf, had the highest yield when pyrolyzed at 350 °C for 0.5 h. B-biochar claimed 51.7%, while L-biochar reported 53.9%. The lowest yields were 27.4% and 28.6% for B-biochar and L-biochar, respectively. They both occurred at 750 °C for 1 h.

### 3.2. The pH and EC

Pyrolysis temperature was determinant for the increase of pH and EC of pond cypress-based biochar. Raw materials from the branch part featured a pH of 3.93, and the pH for the leaf part was 3.90 (Table 1). After pyrolyzing at 750 °C for 2 h, the pH of B-biochar increased to 11.2. L-biochar reached a pH of 12.8 with the pyrolysis at 750 °C for 0.5 h, the highest of all manufactured biochar (Figure 1c,d). However, the ANOVA analysis showed an interactive effect from pyrolysis temperature and time existed on their pH. Moreover, according to Figure 1c,d, the pH of both B-biochar and L-biochar varied significantly between durations when pyrolyzed at 650 and 750 °C, which contrasted with the mild variation with time at lower temperatures like 350 and 450 °C. The pH of B-biochar started to increase drastically on the temporal scale when the temperature hit 650 °C. At 750 °C, its pH increased by 1.6 as the residence time increased from 0.5 to 2 h. For L-biochar, its increasing trend in pH with the time also got pronounced at 650 °C but turned out to be a drastic decreasing trend at 750 °C. This opposite trend with time may be ascribed to the disparity in biomass between different parts of the pond cypress tree. Furthermore, extending the residence time did not always result in higher alkalinity of the biochar with its pyrolysis temperature ≥700 °C. Likewise, the electrical conductivity (EC) of all biochar considerably increased with the temperature. However, the EC of pond cypress-based biochar got little affected by the duration when the pyrolysis temperature held constant. The only exception happened on B-biochar pyrolyzed at 650 °C, which witnessed a big jump in its EC from 0.4 to 1.1 ms·cm-1 as residence time increased from 0.5 to 1 h. On the other hand, the EC of L-biochar flattened with time at four temperature groups in this paper. (Figure 1e,f). This was in line with the result of the ANOVA analysis in that there was no interactive effect of pyrolysis temperature and time on the EC of L-biochar.

### 3.3. The Specific Surface Area (SSA) of Biochar

A typical property of biochar is its massive specific surface area, positively correlated with the absorption capacity of pollutants [2,6,17,18,19]. The larger the surface area, the more favorable conditions exist for microbial activity, enhancing soil fertility and water holding capacity [12,16]. The specific surface area (SSA) of biochar can expand via pyrolysis. There was no exception to biochar from pond cypress (*Taxodium ascendens*), featuring a steep increasing line in their SSA on the temperature scale (Figure 1g,h). The SSA of L-biochar produced at 350 °C for 1 h bottomed 99 m^2^·g^−1^ and peaked at 546 m^2^·g^−1^ with the pyrolysis at 650 °C for 2 h. Moreover, the B-biochar produced at 750 °C for 1 h featured an SSA of 744 m^2^·g^−1^, six times the SSA of 124 m^2^·g^−1^ at 350 °C for 0.5 h. An increasing trend on the temporal scale was also observed in biochar SSA when the temperature was below or equaled 650 °C. Nonetheless, the SSA of B-biochar and L-biochar fluctuated over time as the temperature hit 750 °C, suggesting a greater specific surface area of biochar can achieve by extending the duration within the temperature range from 350 to 650 °C. Besides, pyrolysis time played a more significant role in the SSA of B-biochar at higher temperatures (Figure 1g), indicating an interactive effect between temperature and time.

### 3.4. Proximate Analysis

As presented in Table 2, the moisture content for B-biochar was about 2–8%, while L-biochar claimed 3–6%. Volatile matters (VM) content for all biochar presented a decaying trend with increasing temperature and holding hours. Both B-biochar and L-biochar experienced a more considerable decline in VM contents when temperature increased from 450 to 650 °C. Moreover, volatiles reduced significantly with increasing pyrolysis time in this temperature range. In contrast, fixed carbon (FC) content for B-biochar increased from 25% to 74%, while that for L-biochar increased from 41% to 55%. However, the FC concentration of B-biochar did not necessarily rise or decrease with time. In the case of L-biochar, a significant rise in FC content due to longer duration appeared at higher temperatures, such as 650 °C and 750 °C. Ash content for B-biochar initially increased from 4.41% to 6.28% when temperature climbed from 350 to 450 °C, but decreased to 2.34% at 650 °C and to 2.47% at 750 °C. On the other hand, the ash content for L-biochar increased from 10% to 17% as pyrolysis temperature and time increased. At 650 and 750 °C, L-biochar had a significantly higher ash concentration, almost 3–4 times that of B-biochar.

### 3.5. Elemental Composition Analysis

#### 3.5.1. Ultimate Analysis

Dehydration and deoxygenation during pyrolysis lead to the removal of H and O over C, resulting in the accumulation of C in solid residual biochar. Therefore, discussion about variations in biochar’s C, H, O, and N contents as well as H/C, and O/C molar ratios can shed light on how structures and compositions for manufactured biochar changes during thermal treatment. As we can see from Table 3, all biochar, no matter from branch or leaf part, followed the same trend when the temperature increased from 350 to 750 °C. On one hand, the C content for B-biochar increased from 64.06–67.48% to 70.15–80.52%, and L-biochar had a C content of 61.12–61.24% to 65.87–70.48%. On the other hand, the H and O content decreased consistently with increasing temperature. Compared to pyrolysis temperature’s significant effect on the C, H, and O contents of biochar, heating duration’s effect seemed to be negligible when temperature was controlled, especially for O and H content. Furthermore, no significant variations in the N content of any sampled biochar were seen as the temperature climbed from room temperature to 350 °C (Table 3). The N content of L-biochar averaged 1.88% under varied heating settings in this study, while B-biochar averaged 0.66%. Such a discrepancy in amount was due to the initial N content in raw materials. According to Table 1, the leaf-based biomass had a N content of about 1.42%, whereas that for branch-based feedstock was 0.31%.

**Figure 2:**
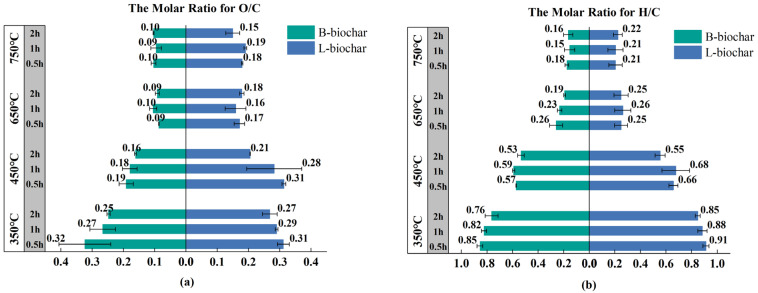
Illustrates how the molar ratios for O/C and H/C of all manufactured biochar evolved with the pyrolysis temperature and time: (**a**) indicates comparison of the O/C molar ratio between B-biochar and L-biochar; while (**b**) indicates comparison of the H/C ratio between B-biochar and L-biochar.

The decrease in the O/C molar ratio as a function of pyrolysis temperature was the same between B-biochar and L-biochar. At 350 °C and 450 °C, the O/C ratio for all biochar presented a consistent decreasing trend with time and remained steady between durations when the temperature hit 650 °C and 750 °C (Figure 2a). As for the H/C molar ratio (Figure 2b), both B-biochar and L-biochar revealed a steep drop as the temperature increased from 350 to 650 °C, where the ratio also fell with increasing duration hours in general. At 750 °C, no notable changes between durations existed. The ratio of B-biochar was between 0.15 and 0.18, while that for L-biochar was between 0.21 and 0.22.

#### 3.5.2. Elements Composition on Biochar Surface

Table 4 shows how the elemental makeup of the biochar surface layer varied depending on the pyrolysis conditions. Compared to other elements, the Ca content was the highest on the surface layer. The content of Ca in L-biochar increased with temperature and then fell, reaching a maximum of about 4.36% after 1 h of pyrolysis at 650 °C; the content of Ca in B-biochar followed the same trend, peaking at 3.48% after 0.5 h of pyrolysis at 650 °C. K had the second highest content. The K contents of B-biochar pyrolyzed at 650 °C for 0.5 h (1.85%) and 750 °C for 2 h (1.65%) were significantly greater than that (less than 1%) of B-biochar produced under other pyrolysis settings. For L-biochar, the overall K content on the surface saw a trend of dropping and then increasing under various pyrolysis settings, reaching the maximum value of 2.65% at 750 °C for 2 h. The content of other elements was modest, and temperature and duration have little effect on their content. However, the Mg content of L-biochar was comparatively higher, with a mean value of 0.69%, much higher than the 0.17% for B-biochar.

### 3.6. Comprehensive Evaluation for 24 Types of Biochar

#### 3.6.1. Principal Component Analysis

Principal component analysis was done to give more comprehensive and in-depth information about features of biochar generated from pond cypress branches and leaves with varied pyrolysis settings. All used data came from the proximate analysis, ultimate analysis, EDS measurement, pH measurement, and EC measurement. Bartlett’s test of sphericity and the Kaiser-Meyer-Olkin measure of sample adequacy indicated that the data were acceptable and suitable for factor analysis, with significance levels of 0.774 and 0.000, respectively. The results revealed that all biochar property indicators were primarily classified into three components, explaining 84.57% of the total variance. The contents of Mg, P, S, Ca, and K on the surface of biochar, as well as the contents of N and Ash, pH, and EC were classified into principal component 1(PC1), which accounted for 37.61% of the overall variation. The principal component 2 (PC2) mainly consists of the contents for C, H, O, fixed carbon, volatile matters, as well as the molar ratios for H/C, and O/C, explaining 34.77% of the total variances. The contribution of Principal component 3(PC3) was 12.19%, including Na and Si contents on biochar surface.

Based on the three principal components, Table 5 gives the component scores of all manufactured biochar. PC1 properties are associated with biochar’s alkalinity and nutrient supplements, which favors in neutralizing acidic soil or enriching nutrients in soil environment. In PC1, B-biochar and L-biochar both scored maximum value at 750 °C for 2 h, with the former being-0.15 and the latter 3.66. L-biochar may be more helpful for treating acidic soil and supplying more nutrients. PC2 attributes represent biochar’s aromaticity, dictating the stability of the C structure in biochar. All biochar had an increasing score in PC2 with the temperature despite the fluctuating trend with time. It is worth saying that B-biochar and L-biochar produced at 650 °C for each duration setting in this experiment enjoyed a comparatively higher score in PC2, suggesting that 650 °C could be the best temperature range for biochar from pond cypress (*Taxodium Ascendens*) to form a more stable C structure. Lastly, the score for PC3 (Na & Si contents on the surface layer of biochar) increased with temperature. In general, there was a positive correlation for B-biochar between the score and pyrolysis time when the temperature hit 350 °C, 450 °C, and 750 °C. Meanwhile, the score for L-biochar fluctuated with time at every temperature setting.

#### 3.6.2. Cluster Analysis

**Figure 3:**
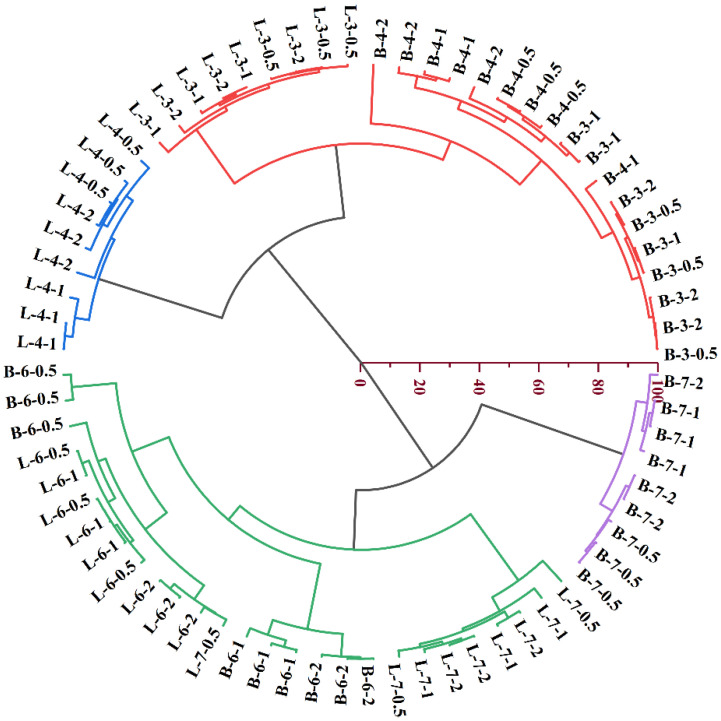
Illustrates that 24 types of manufactured biochar (three replicates for each biochar type) have been classified into 4 groups (different colors represents different groups) by cluster analysis B or L here means raw materials came from branch or leaf part; 350, 450, 650 and 750 represents the temperature for biochar manufacturing; and 0.5, 1, and 2 means residence hours.

Based on the results of principal component analysis, together with the yield and SSA of all biochar sample described above, cluster analysis was done on 24 biochar types. Just as demonstrated in Figure 3, four categories can be divided. The B-biochar and L-biochar made at 350 °C, with B-biochar produced at 450 °C, fell into the first category. L-biochar generated at 450 °C came into the second category. The third category mainly included the B-biochar and L-biochar manufactured at 650 °C, as well as the L-biochar produced at 750 °C. All B-biochar made at 750 °C composed the fourth group. The result indicated that significant differences existed between biochar made from branch and leaf parts of pond cypress (*Taxodium Ascendens*) when the temperature equaled or exceeded 450 °C.

## 4. Discussion

### 4.1. Pyrolysis Time Can Play a Significant Role in Biochar Modification at Specific Temperatures

Pyrolysis temperature is determinant for biochar modification, but residence time can play a significant role at specific temperature. Firstly, it is not hard to find out that both B-biochar and L-biochar featured a significant variation in their pH values when pyrolyzed at 650 and 750 °C (Figure 1c,d), which gets supported by the finding that biochar alkalinity may get enhanced when pyrolyzed at ≥500 °C [22]. That is because dehydration and decarboxylation take place during the biochar pyrolysis process, the former mainly occurs below 500 °C [29,34], and the latter becomes increasingly enhanced over 400 °C [34], both of which cause hydrogen ions (H^+^) to evaporate as water, resulting in a reduction of acidic functional groups on the surface of biochar. Therefore, 650 and 750 °C may be the target temperatures where the acidic functional groups on biochar surface drop sharply and altering the length of residence time at these temperatures may significantly affect the pH value of resulting biochar. In addition, the pH of L-biochar did not necessarily increase with pyrolysis time, especially when pyrolyzed at higher temperature (750 °C) (Figure 1d). That is inconsistent with the finding of A. Shaaban 2014 that the pH value of biochar derived from rubber wood sawdust increased with the rising temperature (300–700 °C) and holding time (1–3 h) [29]. This inconsistency may ascribe to a disparity in whether the raw material was more wood-based or foliage-based. After all, B-biochar (derived from branch part) featured increasing alkalinity as the duration prolonged at four temperature groups (Figure 1c).

Secondly, pyrolysis temperature was the primary driver of the biochar yield [7,15,22], but for B-biochar produced in this study at 350 and 650 °C, particularly 350 °C, changing the length of residence time can have a significant impact, even though this phenomenon didn’t exist for the L-biochar yield (Figure 1a,b). Several reasons account for this phenomenon. For one thing, the biochar yield is closely related to the weight loss of biomass during its pyrolysis in the temperature range from 100 to 400 °C [33], where the loss of water and initial dehydration reactions of the wood and grass precursors happened [7,33,35]. For another, the lignocellulose decomposition begins at 350 °C and approaches the end at a temperature of about 700 °C [7,36,37]. Relevant studies demonstrated that significant correlations exited between the yield and results of proximate analysis (including the content of ash, volatile matters and fixed carbon) [15,22,26,33]. Table 2 showed that the ash and VM contents changed significantly when the temperature increased from 350 to 450 °C; and the ash, VM and FC all presented significant variations as the temperature increased from 450 to 650 °C. All of the abovementioned evidence points to 350 and 650 °C as the key temperatures where the carbon yield changes considerably, so how long biochar would be pyrolyzed at these temperatures can affect their yield in the end.

Thirdly, the SSA of B-biochar saw a drastic change between durations when temperature hit 650 and 750 °C. High temperature facilitates the increase of the specific surface area of biochar, and the SSA usually enjoys an exponential increase when the pyrolysis temperature increased to a high temperature (≥500 °C) [38,39,40]. This phenomenon can be explained by the fact that chemicals, such as ethylene and esters, gradually dissipate from biomass’s outer surface as the temperature of the heating process rises (usually from 300 to 800 °C), favoring the formation of pore structure and the SSA of biochar [38,39,40]. In the same situation as above, such an exponential increase tends to result in a significant variation for the SSA of biochar between durations. However, it is worth noting that the SSA of B-biochar and L-biochar fluctuated with time as the temperature hit 750 °C, and even an obvious decrease can be found (Figure 1g,h).The possible reason for this is high temperature may also induce deformation and collapse of some fine-pore structure, increasing the pore size [38,39,40]. These broken pores are likely to be overcovered by by-products of previous physiochemical reactions, such as incompletely volatilized bio-oil or oxides created by further decomposing carbonates produced following decarboxylation, ultimately lowering the specific surface area [23,24,26].

Given that the physicochemical processes involved in char-making vary at various temperatures, the length of duration at the desired temperature can affect whether these processes start, finish, or proceed adequately. Therefore, in the actual biochar-making, the residence time affecting the resulting biochar characteristics was subject to the pyrolysis temperature setting.

### 4.2. Significant Differences Existed between B-Biochar and L-Biochar Even Though They Were Made from the Same Tree Species

The biochar manufactured in this paper came from different parts of the same tree species. Nonetheless, significant differences existed between B-biochar and L-biochar made at the same pyrolysis conditions. Analyses above have shown that L-biochar featured an obviously higher value in pH and EC (Figure 1c–f). Considering the positive correlation between ash content and the pH and EC of biochar [22,23,29], the far higher ash content in L-biochar can factor for it (Table 2). Additionally, the higher Mg content on the surface of L-biochar (Table 4) can contribute to the higher alkalinity. Studies have proven that increasing the calcium (Ca) and magnesium (Mg) content on biochar’s surface layer promotes its alkalinity and capacity for ion exchange [18,22,41].

Besides, the cluster analysis (Figure 3) revealed that the temperature range of 450–750 °C is a stage at which pyrolysis significantly alters the comprehensive characteristics of B-biochar and L-biochar. The first and foremost reason for such a result can be ascribed to disparities in biomass. As can be seen from Table 1, branch-based raw materials had a cellulose content of 29.11%, whereas the leaf part had a cellulose level of 15.77%. Another difference was the N content, 0.31% for the leaf part while 1.42% for the branch part. The feedstock type is the fundamental. However, the second reason can explain why significant differences appear when biochar pyrolyzed at the temperature range of 450–750 °C. That is most thermochemical reactions happen during this stage. First, thermogravimetric analysis (TGA)shows that the dehydration of biomass mainly occurs at 100–400 °C [3,29,42], leading to a drastic decline in biochar yield at around 350 °C (Figure 1a,b). Previous discussion has pointed the close relation between the biochar yield and the ash content that is positively correlated with the N content (Pearson’s r = 0.805, *p* < 0.01). Moreover, the cellulose degrades at the temperature range of 315–400 °C, giving rise to variations in the molar ratio of O/C and H/C, indicators of aromatic structures in biochar [15,36,37]. In addition, the aromatic C structure starts to form when the temperature hits 400 °C and gets promoted at the temperature range of 600–800 °C [34,35]. Consequently, the distinct discrepancy in cellulose content can be observed in the stability of biochar pyrolyzed during this temperature range. This was verified by the scores of PC2 (about the aromatic degree of biochar) for B-biochar and L-biochar. The former averagely scored −0.18 at 350 °C while the latter scored −0.59. As the temperature increased from 450 to 750 °C, B-biochar had an average score for PC2 was 0.39, whereas that for L-biochar was 0.15 (Table 5).

### 4.3. Biochar Made from Pond Cypress (Taxodium Ascendens) has Advantages on Soil Amendment and Carbon Sequestration

Most of the biochar made from agricultural and forestry wastes has a high pH and EC value, which makes it a feasible additive to deal with soil acidization. A higher EC value means higher cation exchange capacity, favoring the transportation of nutrients like N, P, K, Ca, and Mg [11,12,22]. However, the feedstock type is one of the determining factors for the pH and EC of resulting biochar. With the same or similar pyrolysis conditions as in this paper, the mean value for the pH of biochar made from oak wood, pine wood, spruce wood, poplar wood, Douglas fir wood, and rubber wood was, respectively, 8.76, 7.75, 8.06, 8.97, 8.67, and 8.85 [22,23,29,34]. Meanwhile, the average EC of oak-biochar was claimed to be 0.09 ms·cm^−1^, pine-biochar 0.09 ms·cm^−1^, spruce-biochar 0.98 ms·cm^−1^, as well as poplar-biochar 0.87 ms·cm^−^^1^. In contrast, B-biochar and L-biochar averaged the pH value of about 9.07 and 10.56, and the EC value of about 0.88 and 2.00 ms·cm^−1^ respectively (Figure 1c–f). Therefore, biochar obtained from pond cypress (*Taxodium Ascendens*) may have advantages in soil deacidification and soil fertility enhancement.

Biochar implementation has also been hailed as a feasible and profitable strategy for carbon sequestration in global forestlands owing to its stable C structure [12,13,14,15,16]. There is a definite statement that the most stable black C form exists in biochar with the molar ratio of O/C less than 0.2, enjoying an estimated half-life of more than 1000 years; biochar has an intermediate half-life (100–1000 years) when its O/C ratio ranges from 0.2 to 0.6 [15,43]. In Table 3, B-biochar obtained above 350 °C featured an O/C ratio of 0.09–0.19, and L-biochar produced above 450 °C featured an O/C ratio of 0.15–0.19, both of which would have a half-life of more than 1000 years. Moreover, under the temperature from 650 to 750 °C, the H/C ratio for B-biochar averaged 0.19, while that for L-biochar was 0.17. This indicates that a more fused aromatic ring structure has formed, which benefits the resistance of biochar to microbial and chemical degradation [15,44,45].

## 5. Conclusions

In conclusion, most sampled biochar had a higher pH, EC value, a lower molar ratio of H/C and O/C, and the maximum SSA of 744 m^2^·g^−1^ went to the B-biochar made at 750 °C for 1 h. Biochar from branch and leaf part of the same tree species can possess significantly different properties. How well residence time affected biochar modification was limited by the heating temperature and raw materials.

Besides, several recommendations for biochar production are as follows. Slow pyrolysis (≥2 h) at a higher temperature (≥500 °C) can improve the pH of branch-based biochar from the pond cypress. With the pyrolysis temperature (≤650 °C), extending the residence time can increase the SSA of the produced biochar. With the pond cypress as the source material, pyrolysis at 650 °C for 0.5 h can be the optimal option to attain biochar with a stable C structure. However, some limitations are worth noting. The duration sequence should be extended and refined at the target temperature to get more information for pyrolysis time optimization. Further, a more systematic comparison of biochar properties between coniferous and broadleaf species is needed in the future to provide a holistic and integrated picture of the effects of raw material types.

## Figures and Tables

**Table 1:** Characteristics of Raw Materials.

Compositions	Content (wt.%)	
	Brach	Leaf
Lignin	33.51	39.44
Cellulose	29.11	15.17
Semi-cellulose	12.49	10.03
C	47.70	47.20
H	6.88	6.18
N	0.31	1.42
O	46.79	40.00
Volatile matters	31.43	29.42
Ash	3.16	2.06
pH	3.93	3.90
EC (ms·cm^−1^)	0.32	1.22

**Table 2:** Proximate analysis for all biochar.

Tempt (°C)	Time (h)	Proximate Analysis (%) n = 3			
		Moisture	VM	Ash	FC
Branch					
350	0.50	2.04 ± 0.00	76.56 ± 0.30	4.24 ± 0.10	17.16 ± 0.30
350	1.00	1.92 ± 0.10	71.45 ± 2.00	4.69 ± 0.10	21.94 ± 2.00
350	2.00	2.09 ± 0.00	56.94 ± 0.10	4.32 ± 0.00	36.66 ± 0.10
450	0.50	3.15 ± 0.10	52.00 ± 0.10	5.94 ± 0.10	38.91 ± 0.10
450	1.00	2.54 ± 0.30	53.49 ± 0.10	5.93 ± 0.10	38.04 ± 0.30
450	2.00	3.50 ± 0.30	54.63 ± 0.00	6.96 ± 0.10	34.91 ± 0.30
650	0.50	1.35 ± 0.00	30.35 ± 0.00	2.92 ± 0.10	65.38 ± 0.10
650	1.00	2.68 ± 0.10	23.87 ± 0.00	2.32 ± 0.00	71.13 ± 0.10
650	2.00	5.88 ± 0.10	23.82 ± 0.10	1.77 ± 0.40	68.53 ± 0.60
750	0.50	6.24 ± 0.10	22.56 ± 0.10	1.06 ± 0.00	70.13 ± 0.10
750	1.00	8.58 ± 0.00	17.41 ± 0.10	3.46 ± 0.00	70.55 ± 0.10
750	2.00	8.01 ± 0.20	6.80 ± 0.10	2.89 ± 0.10	82.30 ± 0.20
Leaf					
350	0.50	3.16 ± 0.10	46.19 ± 0.00	10.05 ± 0.10	40.61 ± 0.10
350	1.00	3.46 ± 0.10	45.00 ± 0.10	10.13 ± 0.10	41.42 ± 0.10
350	2.00	3.27 ± 0.10	44.82 ± 0.10	10.32 ± 0.40	41.60 ± 0.30
450	0.50	4.27 ± 0.10	44.60 ± 0.00	11.00 ± 0.10	40.13 ± 0.10
450	1.00	4.67 ± 0.20	43.75 ± 0.10	13.63 ± 0.10	37.95 ± 0.10
450	2.00	4.88 ± 0.20	42.52 ± 0.00	13.47 ± 0.40	39.14 ± 0.50
650	0.50	3.03 ± 0.10	37.06 ± 1.40	12.43 ± 0.10	47.49 ± 1.60
650	1.00	2.99 ± 0.20	29.55 ± 1.20	12.38 ± 0.10	55.08 ± 1.40
650	2.00	4.68 ± 0.20	23.94 ± 0.10	15.73 ± 0.10	55.64 ± 0.20
750	0.50	5.20 ± 0.20	22.79 ± 0.60	15.88 ± 0.10	56.12 ± 0.80
750	1.00	5.36 ± 0.30	20.95 ± 0.00	17.43 ± 0.40	56.26 ± 0.60
750	2.00	6.82 ± 0.20	13.89 ± 0.00	17.06 ± 0.20	62.23 ± 0.30

VM means Volatile Matter, FC means Fixed Carbon, and Tempt means temperature.

**Table 3:** Elemental composition for all biochar.

Tempt (°C)	Time (h)	Ultimate Analysis (%) n = 2			
		C	H	N	O
Branch					
350	0.50	64.06 ± 2.52	4.56 ± 0.06	0.63 ± 0.17	29.07 ± 0.06
350	1.00	65.26 ± 0.58	4.47 ± 0.07	0.69 ± 0.11	27.10 ± 0.04
350	2.00	67.48 ± 0.38	4.30 ± 0.26	0.59 ± 0.07	24.24 ± 0.06
450	0.50	69.63 ± 0.83	3.32 ± 0.03	0.69 ± 0.13	20.01 ± 0.20
450	1.00	69.33 ± 1.51	3.42 ± 0.02	0.66 ± 0.08	20.00 ± 0.05
450	2.00	71.32 ± 0.28	3.17 ± 0.14	0.72 ± 0.09	18.1 ± 0.07
650	0.50	84.33 ± 5.43	1.81 ± 0.24	0.66 ± 0.10	11.06 ± 0.11
650	1.00	74.29 ± 4.50	1.44 ± 0.20	0.48 ± 0.23	10.12 ± 0.08
650	2.00	81.90 ± 4.33	1.37 ± 0.09	0.67 ± 0.06	11.44 ± 0.08
750	0.50	70.15 ± 1.44	1.03 ± 0.10	0.55 ± 0.08	10.47 ± 0.08
750	1.00	80.52 ± 3.33	0.83 ± 0.16	0.59 ± 0.08	10.63 ± 0.1
750	2.00	73.51 ± 2.84	1.00 ± 0.18	0.41 ± 0.18	10.61 ± 0.28
Leaf					
350	0.50	61.14 ± 1.0	4.65 ± 0.04	2.14 ± 0.05	26.17 ± 0.04
350	1.00	61.17 ± 1.41	4.50 ± 0.08	2.15 ± 0.05	25.55 ± 0.61
350	2.00	61.24 ± 1.56	4.33 ± 0.01	2.09 ± 0.00	26.29 ± 0.10
450	0.50	62.64 ± 1.54	3.43 ± 0.1	1.98 ± 0.03	23.34 ± 0.04
450	1.00	67.12 ± 1.98	3.80 ± 0.94	2.35 ± 0.50	24.38 ± 0.10
450	2.00	65.7 ± 2.41	3.04 ± 0.32	1.77 ± 0.20	22.45 ± 0.05
650	0.50	69.55 ± 0.54	1.44 ± 0.30	1.87 ± 0.05	17.22 ± 0.05
650	1.00	67.66 ± 2.68	1.46 ± 0.21	1.61 ± 0.10	15.84 ± 0.37
650	2.00	67.43 ± 2.16	1.39 ± 0.27	1.52 ± 0.10	15.88 ± 0.05
750	0.50	70.48 ± 2.18	1.20 ± 0.27	1.99 ± 0.00	17.67 ± 0.06
750	1.00	65.87 ± 3.48	1.11 ± 0.20	1.49 ± 0.15	16.91 ± 0.09
750	2.00	66.51 ± 0.54	1.24 ± 0.20	1.68 ± 0.08	17.28 ± 0.06

Tempt means temperature.

**Table 4:** Elemental composition on biochar surface.

Tempt (°C)	Time (h)	Elemental Content Measured By EDS (wt%) n = 3						
		S	P	K	Ca	Na	Mg	Si
Branch								
350	0.50	0.17 ± 0.00	0.11 ± 0.00	0.30 ± 0.10	0.94 ± 0.00	0.23 ± 0.00	0.08 ± 0.00	0.16 ± 0.00
350	1.00	0.22 ± 0.00	0.12 ± 0.00	0.53 ± 0.10	1.07 ± 0.80	0.14 ± 0.00	0.14 ± 0.00	0.18 ± 0.00
350	2.00	0.13 ± 0.00	0.13 ± 0.00	0.47 ± 0.00	0.82 ± 0.40	0.17 ± 0.00	0.05 ± 0.00	0.14 ± 0.00
450	0.50	0.16 ± 0.10	0.03 ± 0.00	0.66 ± 0.00	1.77 ± 0.40	0.11 ± 0.00	0.16 ± 0.00	0.21 ± 0.00
450	1.00	0.19 ± 0.00	0.04 ± 0.00	0.62 ± 0.00	1.64 ± 0.40	0.13 ± 0.00	0.17 ± 0.00	0.15 ± 0.00
450	2.00	0.28 ± 0.10	0.06 ± 0.00	0.60 ± 0.00	1.96 ± 0.30	0.1 ± 0.00	0.22 ± 0.00	0.17 ± 0.00
650	0.50	0.32 ± 0.10	0.25 ± 0.10	1.89 ± 1.40	3.49 ± 1.20	0.1 ± 0.00	0.19 ± 0.00	0.31 ± 0.00
650	1.00	0.3 ± 0.10	0.11 ± 0.10	0.71 ± 0.10	2.4 ± 0.80	0.12 ± 0.00	0.21 ± 0.00	0.36 ± 0.00
650	2.00	0.4 ± 0.00	0.18 ± 0.00	0.64 ± 0.20	2.03 ± 0.90	0.12 ± 0.00	0.23 ± 0.00	0.37 ± 0.00
750	0.50	0.26 ± 0.10	0.1 ± 0.00	0.49 ± 0.10	2.05 ± 0.70	0.23 ± 0.10	0.19 ± 0.00	0.17 ± 0.00
750	1.00	0.31 ± 0.00	0.13 ± 0.00	0.47 ± 0.10	1.63 ± 0.80	0.13 ± 0.00	0.19 ± 0.00	0.15 ± 0.00
750	2.00	0.36 ± 0.10	0.15 ± 0.00	1.65 ± 1.70	2.98 ± 0.70	0.17 ± 0.00	0.22 ± 0.00	0.11 ± 0.00
Leaf								
350	0.50	0.28 ± 0.10	0.22 ± 0.00	1.52 ± 0.10	2.08 ± 0.20	0.21 ± 0.00	0.53 ± 0.00	0.03 ± 0.00
350	1.00	0.37 ± 0.00	0.24 ± 0.00	1.77 ± 0.10	2.44 ± 0.20	0.28 ± 0.10	0.63 ± 0.10	0.04 ± 0.00
350	2.00	0.34 ± 0.00	0.28 ± 0.00	1.61 ± 0.00	2.22 ± 0.00	0.20 ± 0.00	0.47 ± 0.10	0.06 ± 0.00
450	0.50	0.28 ± 0.00	0.24 ± 0.00	1.40 ± 0.30	2.83 ± 0.00	0.20 ± 0.00	0.53 ± 0.00	0.1 ± 0.00
450	1.00	0.31 ± 0.00	0.34 ± 0.10	0.92 ± 0.10	2.81 ± 0.00	0.18 ± 0.00	0.48 ± 0.00	0.13 ± 0.00
450	2.00	0.32 ± 0.00	0.27 ± 0.00	0.88 ± 0.20	2.78 ± 0.00	0.18 ± 0.00	0.57 ± 0.00	0.09 ± 0.00
650	0.50	0.41 ± 0.00	0.4 ± 0.00	1.55 ± 0.50	3.72 ± 0.30	0.15 ± 0.00	0.87 ± 0.00	0.30 ± 0.00
650	1.00	0.39 ± 0.00	0.42 ± 0.00	0.92 ± 0.20	4.36 ± 0.30	0.14 ± 0.00	0.87 ± 0.00	0.31 ± 0.00
650	2.00	0.47 ± 0.10	0.39 ± 0.00	0.81 ± 0.30	4.03 ± 0.00	0.14 ± 0.00	0.79 ± 0.10	0.31 ± 0.00
750	0.50	0.62 ± 0.00	0.44 ± 0.00	2.30 ± 0.00	3.51 ± 0.10	0.16 ± 0.00	0.86 ± 0.00	0.26 ± 0.10
750	1.00	0.53 ± 0.10	0.50 ± 0.00	1.90 ± 0.40	3.02 ± 0.40	0.2 ± 0.00	0.79 ± 0.10	0.17 ± 0.00
750	2.00	0.67 ± 0.10	0.50 ± 0.00	2.65 ± 0.40	3.74 ± 0.30	0.17 ± 0.00	0.88 ± 0.00	0.19 ± 0.00

Tempt means temperature.

**Table 5:** Scores in three principal components for 24 types of biochar.

PC	Pyrolysis Temperature and Durations for Biochar Manufacturing											
	350 °C			450 °C			650 °C			750 °C		
	0.5 h	1 h	2 h	0.5 h	1 h	2 h	0.5 h	1 h	2 h	0.5 h	1 h	2 h
Branch												
PC1	−2.79	−2.54	−2.55	−1.99	−1.79	−1.44	−0.33	−1.09	−0.72	−0.93	−0.54	−0.15
PC2	−0.53	0.11	−0.12	0.58	0.08	0.33	1.45	0.94	1.18	−0.81	0.31	−0.52
PC3	−2.20	−1.99	−1.90	−1.04	−1.10	−0.79	0.68	−0.11	0.18	−0.17	0.01	0.04
Leaf												
PC1	−0.65	−0.13	−0.08	0.36	0.62	0.60	1.84	1.50	1.97	3.19	3.26	3.66
PC2	−0.28	−0.90	−0.61	−0.35	−0.33	−0.71	0.47	0.42	0.24	0.38	−0.86	−0.51
PC3	0.13	0.40	−0.04	0.49	0.43	0.11	1.25	1.05	0.86	1.66	0.81	1.26

PC here means principal components.

## References

[1] Masek O., Buss W., Brownsort P., Rovere M., Tagliaferro A., Zhao L., Cao X.D., Xu G.W. (2019). Potassium doping increases biochar carbon sequestration potential by 45%, facilitating decoupling of carbon sequestration from soil improvement. Sci. Rep..

[2] Zhao B., O’Connor D., Shen Z., Tsang D.C.W., Rinklebe J., Hou D. (2020). Sulfur-modified biochar as a soil amendment to stabilize mercury pollution: An accelerated simulation of long-term aging effects. Environ. Pollut..

[3] Wang Z., Guo H., Shen F., Yang G., Zhang Y., Zeng Y., Wang L., Xiao H., Deng S. (2015). Biochar produced from oak sawdust by Lanthanum (La)-involved pyrolysis for adsorption of ammonium (NH_4_(+)), nitrate (NO_3_(−)), and phosphate (PO_4_(^3^−)). Chemosphere.

[4] Zhang Y., Shang Q., Feng D.D., Sun H.L., Wang F.H., Hu Z.C., Cheng Z.Y., Zhou Z.J., Zhao Y.J., Sun S.Z. (2022). Interaction mechanism of in-situ catalytic coal H_2_O-gasification over biochar catalysts for H_2_O-H-_2_-tar reforming and active sites conversion. Fuel Process. Technol..

[5] Susastriawan A.A.P., Saptoadi H. (2017). Purnomo, Small-scale downdraft gasifiers for biomass gasification: A review. Renew. Sustain. Energy Rev..

[6] Yang Y., Wei Z., Zhang X., Chen X., Yue D., Yin Q., Xiao L., Yang L. (2014). Biochar from Alternanthera philoxeroides could remove Pb(II) efficiently. Bioresour. Technol..

[7] Wang H., Wang X., Cui Y., Xue Z., Ba Y. (2018). Slow pyrolysis polygeneration of bamboo (*Phyllostachys pubescens*): Product yield prediction and biochar formation mechanism. Bioresour. Technol..

[8] Foong S.Y., Chan Y.H., Chin B.L.F., Lock S.S.M., Yee C.Y., Yiin C.L., Peng W.X., Lam S.S. (2022). Production of biochar from rice straw and its application for wastewater remediation—An overview. Bioresour. Technol..

[9] Li Y.C., Xing B., Ding Y., Han X.H., Wang S.R. (2020). A critical review of the production and advanced utilization of biochar via selective pyrolysis of lignocellulosic biomass. Bioresour. Technol..

[10] Liang M.A., Lu L., He H.J., Li J.X., Zhu Z.Q., Zhu Y.N. (2021). Applications of Biochar and Modified Biochar in Heavy Metal Contaminated Soil: A Descriptive Review. Sustainability.

[11] Shah T., Khan S., Shah Z. (2017). Soil respiration, pH and EC as influenced by biochar. Soil Environ..

[12] Zornoza R., Moreno-Barriga F., Acosta J.A., Muñoz M.A., Faz Á. (2016). Stability, nutrient availability and hydrophobicity of biochars derived from manure, crop residues, and municipal solid waste for their use as soil amendments. Chemosphere.

[13] Windeatt J.H., Ross A.B., Williams P.T., Forster P.M., Nahil M.A., Singh S. (2014). Characteristics of biochars from crop residues: Potential for carbon sequestration and soil amendment. J. Environ. Manag..

[14] Ennis C.J., Evans A., Islam M., Ralebitso-Senior T.K., Senior E. (2012). Biochar: Carbon Sequestration, Land Remediation, and Impacts on Soil Microbiology. Crit. Rev. Environ. Sci. Technol..

[15] Leng L., Huang H. (2018). An overview of the effect of pyrolysis process parameters on biochar stability. Bioresour. Technol..

[16] Lehmann J., Cowie A.L., Masiello C.A., Kammann C., Woolf D., Amonette J.E., Cayuela M.L., Camps-Arbestain M., Whitman T.L. (2021). Biochar in climate change mitigation. Nat. Geosci..

[17] Ahmad M., Rajapaksha A.U., Lim J.E., Zhang M., Bolan N., Mohan D., Vithanage M., Lee S.S., Ok Y.S. (2014). Biochar as a sorbent for contaminant management in soil and water: A review. Chemosphere.

[18] Tan X., Liu Y., Zeng G., Wang X., Hu X., Gu Y., Yang Z. (2015). Application of biochar for the removal of pollutants from aqueous solutions. Chemosphere.

[19] Dai Y., Wang W., Lu L., Yan L., Yu D. (2020). Utilization of biochar for the removal of nitrogen and phosphorus. J. Clean. Prod..

[20] Azzi E.S., Karltun E., Sundberg C. (2021). Assessing the diverse environmental effects of biochar systems: An evaluation framework. J. Environ. Manag..

[21] Al-Rumaihi A., Shahbaz M., McKay G., Mackey H., Al-Ansari T. (2022). A review of pyrolysis technologies and feedstock: A blending approach for plastic and biomass towards optimum biochar yield. Renew. Sustain. Energy Rev..

[22] Zhang H., Chen C., Gray E.M., Boyd S.E. (2017). Effect of feedstock and pyrolysis temperature on properties of biochar governing end use efficacy. Biomass Bioenergy.

[23] Suliman W., Harsh J.B., Abu-Lail N.I., Fortuna A.-M., Dallmeyer I., Garcia-Perez M. (2016). Influence of feedstock source and pyrolysis temperature on biochar bulk and surface properties. Biomass Bioenergy.

[24] Elnour A.Y., Alghyamah A.A., Shaikh H.M., Poulose A.M., Al-Zahrani S.M., Anis A., Al-Wabel M.I. (2019). Effect of Pyrolysis Temperature on Biochar Microstructural Evolution, Physicochemical Characteristics, and Its Influence on Biochar/Polypropylene Composites. Appl. Sci..

[25] Tripathi M., Sahu J.N., Ganesan P.B. (2016). Effect of process parameters on production of biochar from biomass waste through pyrolysis: A review. Renew. Sustain. Energy Rev..

[26] Guedes R.E., Luna A.S., Torres A.R. (2018). Operating parameters for bio-oil production in biomass pyrolysis: A review. J. Anal. Appl. Pyrolysis.

[27] Li S.M., Harris S., Anandhi A., Chen G. (2019). Predicting biochar properties and functions based on feedstock and pyrolysis temperature: A review and data syntheses. J. Clean. Prod..

[28] Mahdi Z., El Hanandeh A., Yu Q.M. (2017). Influence of Pyrolysis Conditions on Surface Characteristics and Methylene Blue Adsorption of Biochar Derived from Date Seed Biomass. Waste Biomass Valorization.

[29] Shaaban A., Se S.-M., Dimin M.F., Juoi J.M., Mohd Husin M.H., Mitan N.M.M. (2014). Influence of heating temperature and holding time on biochars derived from rubber wood sawdust via slow pyrolysis. J. Anal. Appl. Pyrolysis.

[30] Cross A., Sohi S.P. (2013). A method for screening the relative long-term stability of biochar. Glob. Change Biol. Bioenergy.

[31] Zhang J., Liu J., Liu R. (2015). Effects of pyrolysis temperature and heating time on biochar obtained from the pyrolysis of straw and lignosulfonate. Bioresour. Technol..

[32] Lai C., Jia Y., Wang J., Wang R., Zhang Q., Chen L., Shi H., Huang C., Li X., Yong Q. (2019). Co-production of xylooligosaccharides and fermentable sugars from poplar through acetic acid pretreatment followed by poly (ethylene glycol) ether assisted alkali treatment. Bioresour. Technol..

[33] Zhang X., Zhang P., Yuan X., Li Y., Han L. (2020). Effect of pyrolysis temperature and correlation analysis on the yield and physicochemical properties of crop residue biochar. Bioresour. Technol..

[34] Kloss S., Zehetner F., Dellantonio A., Hamid R., Ottner F., Liedtke V., Schwanninger M., Gerzabek M.H., Soja G. (2012). Characterization of slow pyrolysis biochars: Effects of feedstocks and pyrolysis temperature on biochar properties. J. Environ. Qual..

[35] Keiluweit M., Nico P.S., Johnson M.G., Kleber M. (2010). Dynamic Molecular Structure of Plant Biomass-Derived Black Carbon (Biochar). Environ. Sci. Technol..

[36] Yang H., Yan R., Chen H., Lee D.H., Zheng C. (2007). Characteristics of hemicellulose, cellulose and lignin pyrolysis. Fuel.

[37] Paris O., Zollfrank C., Zickler G.A. (2005). Decomposition and carbonisation of wood biopolymers—a microstructural study of softwood pyrolysis. Carbon.

[38] Lian F., Xing B. (2017). Black Carbon (Biochar) In Water/Soil Environments: Molecular Structure, Sorption, Stability, and Potential Risk. Environ. Sci. Technol..

[39] Jia L., Yu Y., Guo J.R., Qin S.N., Wang Y.L., Shen X., Fan B.G., Jin Y. (2020). Study of the Molecular Structure and Elemental Mercury Adsorption Mechanism of Biomass Char. Energy Fuels.

[40] Xiao X., Chen B.L., Chen Z.M., Zhu L.Z., Schnoor J.L. (2018). Insight into Multiple and Multilevel Structures of Biochars and Their Potential Environmental Applications: A Critical Review. Environ. Sci. Technol..

[41] Feng D., Zhao Y., Zhang Y., Zhang Z., Sun S. (2017). Roles and fates of K and Ca species on biochar structure during in-situ tar H_2_O reforming over nascent biochar. Int. J. Hydrog. Energy.

[42] Kocabaş-Ataklı Z.Ö., Okyay-Öner F., Yürüm Y. (2014). Combustion characteristics of Turkish hazelnut shell biomass, lignite coal, and their respective blends via thermogravimetric analysis. J. Therm. Anal. Calorim..

[43] Spokas K.A. (2010). Review of the stability of biochar in soils: Predictability of O:C molar ratios. Carbon Manag..

[44] Kuhlbusch T.A.J. (1995). Method for determining black carbon in residues of vegetation fires. Environ. Sci. Technol..

[45] Han L., Ro K.S., Wang Y., Sun K., Sun H., Libra J.A., Xing B. (2018). Oxidation resistance of biochars as a function of feedstock and pyrolysis condition. Sci. Total Environ..

